# The Prefrontal Cortex and Suggestion: Hypnosis vs. Placebo Effects

**DOI:** 10.3389/fpsyg.2016.00415

**Published:** 2016-03-30

**Authors:** Benjamin A. Parris

**Affiliations:** Department of Psychology, Faculty of Science and Technology, Bournemouth UniversityPoole, UK

**Keywords:** suggestion, hypnosis, placebo, prefrontal cortex (PFC), executive function

Suggestion has been defined as a form of communicable ideation or belief, that once accepted has the capacity to exert profound changes on a person's mood, thoughts, perceptions and behaviors (Halligan and Oakley, 2014). The prefrontal region (the region of the frontal cortex anterior to the motor areas) of the human cerebral cortex appears to play an important role in suggestion (Asp et al., 2012). Children, with still-developing prefrontal cortices, are more susceptible to suggestion (Ceci et al., 1987; Bruck and Ceci, 1995). Older adults, who experience atrophy of the prefrontal cortex (PFC) as a result of the normal processes of aging, are also more open to suggestion (Cohen and Faulkner, 1989). Damasio (1994) described how patients with damage to the PFC are more vulnerable to “snake-oil” salesmen and disreputable characters. Asp et al. (2012) showed that patients with ventromedial PFC damage were more likely to believe in misleading advertisements. The role of the frontal cortices in suggestion fits with the putative role of the prefrontal cortex in the control of thought and behavior (Miller and Cohen, 2001).

Halligan and Oakley (2014) recently called for increased study of phenomena associated with suggestion, noting the importance of suggestion to many forms of human behavior. They compared various forms of suggestion in an attempt to elucidate shared underlying psychological properties. In separate work, they have noted the growing interest in hypnosis and hypnotic suggestion following the application of methods of cognitive neuroscience to the study of these and related phenomena (Oakley and Halligan, 2009). Here the contributions of the prefrontal cortex to hypnosis and placebo effects will be described in an attempt to highlight how methods of cognitive neuroscience might complement other approaches when investigating different forms of suggestion.

Hypnosis involves suggestion. Cardeña and Spiegel (1991) described hypnotic phenomenon as comprising three integrative dimensions, one of which is suggestibility (with the remaining two being absorption and dissociation). Commonly used scales that measure “hypnotisability” are actually measuring suggestibility under hypnosis (Kirsch et al., 2011). Thus, hypnosis is intrinsically linked with suggestion and suggestion is in fact a conditio sine qua non to create and measure hypnotic phenomena.

Hypnosis usually takes the form of an interaction in which one person, the hypnotist, relaxes another individual by talking him/her through a series of visualizations involving muscle relaxation and then counting him/her up into a “state” of hypnosis (known as the induction). The hypnotized individual will be concentrating on the hypnotist's voice relaying a series and variety of suggestions to which the individual may or may not respond. Under the influence of hypnotic suggestion (or post-hypnotic suggestion which is a suggestion given under hypnosis but activated post-hypnosis) the experience of pain (Derbyshire et al., 2004), color perception (Kosslyn et al., 2000), cognitive conflict (Raz et al., 2002) and delusions (Rahmanovic et al., 2011) can be produced or extinguished (see Oakley and Halligan, 2009, for a recent review).

Placebo effects also involve suggestion and are commonly employed in medicine and clinical practice. They are also used as a control condition in randomized controlled trials assessing the clinical significance of new treatments and drugs (Finniss et al., 2010). Comparisons have been drawn between hypnosis and the placebo effect with hypnosis being described as placebo without deception (Wickless and Kirsch, 1989; Kirsch, 1999; Raz, 2007; Kirsch et al., 2008). Raz (2007) made the case that by studying hypnosis and using it as a correlate of placebo one can avoid the ethical considerations associated with deception in placebo, and still understand the mechanisms that underpin it. More specifically, the case was made that the recent finding that a specific COMT polymorphism correlates with hypnotisability indicates that similar associations may tap at least some form of Good Placebo Responder (GPR), and that regardless of the formal relationship between hypnosis and placebo it may be helpful to apply hypnosis for its placebo value in generating positive expectations. A further argument for the association between hypnosis and placebo effects is that they are both influenced by expectancy (Kirsch, 1999), although the case for expectancy as an important determinant appears more convincing in the case of placebos (Benham et al., 1998). Hypnotic suggestibility and response to placebo also both appear to increase following inhalation of oxytocin (Bryant et al., 2013; Kessner et al., 2013; although see Parris et al., 2014 for an example of how oxytocin impedes the effect of a post-hypnotic suggestion), and have similar analgesic effects (Kupers et al., 2005).

Research using Transcranial Magnetic Stimulation (TMS) suggests that placebo effects require the involvement of the prefrontal cortex whereas hypnotic suggestibility is increased when the prefrontal cortex is hypoactive. Applying TMS to the left dorso-lateral prefrontal cortex (DLPFC) to impair function in this region results in increased hypnotic suggestibility (Dienes and Hutton, 2013). In their study, medium suggestible participants received 5 min of 1 Hz repetitive TMS to either the left DLPFC or the vertex (control location) in four separate sessions. In the 5 min of residual cortical disruption that followed stimulation participants were given a brief hypnotic induction and two hypnotic suggestions (magnetic hands and arm levitation). In a second session involving stimulation of the same cortical site, a further two hypnotic suggestions were delivered (rigid arm and taste hallucination). Relative to stimulation of the vertex, low frequency rTMS to the left DLPFC resulted in an increase in hypnotic susceptibility (a 6% increase) to all suggestions as established using both subjective and objective reports of performance. Importantly, their result was not related to expectancy since expectancies were identical between the two conditions. In contrast, Krummenacher et al. (2010) have shown that rTMS to the right and left DLPFC completely blocked expectation-related placebo analgesia. In this study participants received 1 Hz rTMS for 15 min after which heat pain was delivered to their forearms. Compared to a sham condition, those receiving rTMS to the DLPFC did not show placebo analgesic effects (the TMS equipment was used as the placebo) and this was irrespective of the laterality of stimulation. Indeed, the placebo response was completely blocked by inhibition of the DLPFC. The authors hypothesized that the results were due to a disruption of the cognitive representation of pain analgesia and the resulting suppression of an expectation-related placebo effect. In sum, these two studies on two forms of suggestion illustrate a differential and opposing effect of stimulation of the left DLPFC.

There are important differences between these studies that may have a bearing on their outcomes. In Dienes and Hutton's study brain stimulation occurred before the hypnotic induction and suggestions were given, whereas in Krummenacher et al. stimulation occurred after the suggestion of placebo analgesia was given. It is possible that TMS of DLPFC after hypnotic induction and suggestion would also inhibit the effects of suggestion. The effects of TMS might also be dosage dependent: Dienes and Hutton delivered 5 min of stimulation, whereas Krummenacher et al. delivered 15 min. It is possible that had stimulation been delivered for 15 min in the hypnosis study, the effect size would have been larger or the observed effect would have been in the opposite direction. It is also not clear in either case that the result was a consequence of stimulation of the DLPFC since TMS can have indirect effects through synaptic connections. Stimulation of the DLPFC results in activation of the VLPFC (Eisenegger et al., 2008) which has been shown to be activated by surprising and rewarding stimuli (Parris et al., 2007, 2009; Rolls et al., 2008). It has also been shown that rTMS of the DLPFC results in blood flow changes in the ACC and midbrain neurons (Speer et al., 2003).

Despite these issues, the findings from these two studies seem to mirror findings in both literatures. Benedetti (2009) has noted that without PFC there is no placebo effect. Benedetti et al. (2006) studied Alzheimer's patients at the initial stage of the disease and 1-year later to investigate whether Alzheimer's Disease altered susceptibility to placebo effects. They found that the reduced frontal connectivity in patients predicted a smaller placebo response. Stein et al. (2012) have also shown that greater white matter integrity in the DLPFC, rostral anterior cingulate cortex, and the periacaqueductal gray was associated with greater placebo analgesia.

In contrast to Stein et al. a recent study revealed no relationship between white (or gray) matter microstructure and hypnotic suggestibility (Hoeft et al., 2012; although see Huber et al., 2014). However, a relatively well-established finding in the hypnosis literature is that hypnosis itself leads to reduced frontal lobe functioning (Sheehan et al., 1988; Farvolden and Woody, 2004; Jamieson and Sheehan, 2004; Wagstaff et al., 2007), which supports the notion that disrupting frontal activity using TMS would facilitate being hypnotized. One can also consider hypnotic suggestibility itself and whether there is evidence that frontal lobe function is initially relatively impaired in highly suggestible individuals, again facilitating the induction of hypnosis. Consistent with this, studies employing the Stroop task, a task known to involve the DLPFC, report poorer performance by highs at baseline (Dixon et al., 1990; Dixon and Laurence, 1992; Farvolden and Woody, 2004) indicating poorer DLPFC function independent of hypnosis. However, studies with large sample sizes have reported no baseline performance differences between suggestible and less suggestible people on tasks thought to index frontal lobe function (Dienes et al., 2009; Varga et al., 2011), although these latter findings could be due to a failure to titrate task difficulty and involve tasks that do not so clearly rely on DLPFC.

The PFC is a large region of the brain, and one might consider the role of different regions of the PFC in the varieties of suggestion. The involvement of the DLPFC could differentiate hypnosis and placebo effects. The role of more ventromedial regions in other forms of suggestion (Asp et al., 2012) indicates a further potential dissociation between neural substrates underpinning the varieties of suggestion.

Lifshitz et al. (2012) draw a distinction between two types of suggestibility: (1) suggestibility that is largely determined by underlying cognitive aptitude, and (2) suggestibility that is best considered as a flexible skill amenable to attitudinal factors including beliefs and expectations. The differential involvement of the PFC in hypnosis and placebo, and the identification of individual differences in the former and not the latter, appears to indicate that aptitude is most important in hypnosis, whilst attitudinal factors might be more important in the placebo response. Expectancy appears to be a more important determinant in the case of placebos (Benham et al., 1998; Benedetti, 2009; Lifshitz et al., 2012). Indeed, in Dienes and Hutton's TMS study, their effect of TMS on hypnotic suggestibility was independent of expectancy, but Krummenacher et al.'s TMS study reports disruption to an expectancy-based (as opposed to a conditioning-based) placebo.

Notably, research suggests that measures of placebo suggestibility do not correlate with hypnotic/imaginative suggestibility measures (Kihlstrom, 2008) indicating another reason to consider them different entities, at least in some regards. Evidencing a complex relationship between different forms of suggestion, a recent study reported similar levels of placebo analgesia in high and low suggestible individuals despite differential neural activity underpinning it (Huber et al., 2013).

In conclusion, whilst there are potentially informative similarities between hypnotic suggestion and placebos, their differences, particularly with regard to the differential contributions of regions of the prefrontal cortex, are also potentially informative as to the nature of suggestion more generally.

## Author contributions

The author confirms being the sole contributor of this work and approved it for publication.

## Funding

This research was supported by Bournemouth University.

### Conflict of interest statement

The author declares that the research was conducted in the absence of any commercial or financial relationships that could be construed as a potential conflict of interest.
